# Downregulation of Protein Tyrosine Phosphatase Receptor Type R Accounts for the Progression of Hirschsprung Disease

**DOI:** 10.3389/fnmol.2019.00092

**Published:** 2019-04-10

**Authors:** Jiao Tian, Cheng Zeng, Zhen Tian, Yan Lin, Baoxi Wang, Yongkang Pan, Zhen Shu, Xun Jiang

**Affiliations:** ^1^Department of Pediatrics, Tangdu Hospital, The Fourth Military Medical University, Xi’an, China; ^2^Department of Nature Medicine, School of Pharmacy, The Fourth Military Medical University, Xi’an, China; ^3^Department of Pharmacology, School of Pharmacy, Xi’an, China; ^4^Department of Pharmacy and Precision Pharmacy & Drug Development Center, Tangdu Hospital, The Fourth Military Medical University, Xi’an, China; ^5^Department of Neonatal Surgery, The Affiliated Children Hospital of Xi’an Jiaotong University, Xi’an, China; ^6^Department of Radiation Oncology, Winship Cancer Institute, Emory University, Atlanta, GA, United States

**Keywords:** hirschsprung’s disease, enteric neural crest cell, enteric nervous system, protein tyrosine phosphatase receptor-type R, glial cell-line derived neurotrophic factor

## Abstract

Hirschsprung disease (HSCR) is a common developmental disorder of the enteric nervous system (ENS). However, the disease mechanisms have not been fully elucidated. To better understand the etiology of HSCR, the role and mechanism of HSCR associated PTPRR (protein tyrosine phosphatase receptor-type R) in the multipotency of ENS progenitors and ENS development were explored. In the present study, the downregulated PTPRR expression in HSCR was reflected by microarray and validated by real-time PCR analyses. Moreover, PTPRR protein was mainly expressed in the cytoplasmic area of primary cultured ENS progenitors (Enteric neural crest cells, ENCCs) and significantly decreased after differentiation induction, which implies the anti-differentiation role in ENCCs. Further study employed an adenovirus transfection system. After genetic modulation, the ENCCs maintained undifferentiated patterns even in GDNF (Glial cell-line derived neurotrophic factor)-mediated directional differentiation, as well as significantly increased EdU positive immunofluorescence in the PTPRR overexpressing group while the development of the ENS was stunted in the PTPRR knockdown fetal gut. Moreover, the expression of ERK1/2 activated by GDNF was significantly decreased as reflected by western-blot or immunofluorescence analyses after genetic modulation in the PTPRR overexpressing group, which suggests the potential mechanism in regulating the MAPK/ERK1/2 pathway. Taken together, These data support the idea that PTPRR may ensure a certain number of neural precursor cells by inhibiting ENCC overt differentiation and maintaining ENCC proliferation, which is considered to be the multipotency of ENCCs, and eventually participate in the development of the ENS, and establish PTPRR protein as negative regulator of MAPK/ERK1/2 signaling cascades in neuronal differentiation and demonstrate their involvement in the pathophysiology of HSCR.

## Introduction

Hirschsprung disease (HSCR) is the most common identifiable developmental disorder of the enteric nervous system (ENS). The incidence of congenital intestinal diseases in newborns is approximately 1/5000 (Liu et al., 2015; Lin, 2016). Ninety percent of lesions of HSCR locate in the rectum and the distal part of the sigmoid colon. Previous study have identified some susceptibility genes implicated in HSCR, such as *RET*, which is the major gene in HSCR with mutations found in most of the cases and was identified to be crucial for the development of neural crest cells thus involved in the pathogenesis of HSCR (Heanue et al., 2016; Porokuokka et al., 2018). In recent years, new susceptibility genes such as *GLI*, *RELN, GAL*, and *AUTS2* have been found via gene screening (Liu et al., 2015; Wang et al., 2016, 2018; Zhang et al., 2018). This suggests that, genetic variations of HSCR are well-known and have been indentified in < 30% of HSCR cases, indicating the need to identify other variations involving in it (Bahrami et al., 2018).

The ENS is composed of millions of neurons and glial cells that originate from pluripotent stem-like cells, namely, enteric neural crest cells (ENCCs) (Avetisyan et al., 2015). During the course of colonization, ENCCs proliferate and differentiate in overlapping stages to populate the expanding gut and ultimately mediates the normal colonization of ENS. For one hand, proliferation is essential for generating sufficient numbers of ENS progenitor cells. Several signaling pathways and transcriptional factors have been implicated in normal ENCCs proliferation, for example, the Glial cell line-derived neurotrophic factor (GDNF)/ Rearranged during transfection (RET) pathway, Endothelin-3(Et-3)/ Endothelin Receptor B (Ednrb) pathway, and Sry-related high mobility group box10 (Sox10) (Nagy and Goldstein, 2017). For the other hand, ENCC differentiation is another critical process for the development of ENS. Distinct signaling has been identified during subtype differentiation. For example, neurogenesis initiates shortly after vagal ENCCs enter the embryonic foregut and continues till the postnatal stage, which involves the downregulation of Sox10 and p75^NTR^ and maintenance of Ret and Phox2b expression, and the increased expression of neuronal class III β-tubulin (Tuj1), PGP9.5, and HuC/HuD, which are neuronal markers of progressive differentiation (Hao et al., 2010). Gliogenesis commences after neurogenesis is initiated, during which the expressions of Phox2b and Ret are downregulated, with overt expression of the glial marker brain fatty acid binding protein (B-FABP) or the terminal glial differentiation marker glial fibrillary acidic protein (GFAP) (Young et al., 2003). Because GDNF/RET signaling is required for both ENCC proliferation and differentiation, the regulation of GDNF/RET signaling to orchestrate diverse developmental events is of vital significance. However, the exact molecular mechanisms that regulate GDNF/RET signaling levels remain elusive.

In this study, we identified a new specific differentially expressed gene in HSCR. protein tyrosine phosphatase receptor-type R (PTPRR), as a coding gene of the protein tyrosine phosphatase (PTP) subtype, is mainly involved in the negative regulation of protein phosphorylation in the ERK signaling pathway and is closely related to the proliferation and differentiation of neurons (Erkens et al., 2015). Our results suggest that the downregulated expression of the *PTPRR* gene is closely related to HSCR and may be involved in the alterations in the process of gut ENS development through the GDNF-mediated MAPK pathway, thus leading to disease.

## Materials and Methods

### Animal and Clinical Specimens

This study was approved by the Ethics Committee of the Affiliated Children Hospital of Xi’an Jiaotong University (Shaanxi, China) and the guardians of all subjects involved in the study signed written informed consent. Aganglionic and proximal ganglionic tissues were obtained from 33 patients (7 females and 26 males) diagnosed with HSCR at the Neonatal Surgery, the Affiliated Children Hospital of Xi’an Jiaotong University from 01/2014 to 01/2017. The average age of these patients was 25.5 ± 8 days. HSCR was diagnosed in patients, based on symptoms, barium enema, and histochemical evidence of an absence of enteric nervous plexuses of colon tissue. All the patients were diagnosed with short-segment HSCR and without family history of this disease. All specimens were collected immediately after intraoperative resection, washed with sterile saline, and frozen in liquid nitrogen, then stored overnight in -80°C refrigerator for long-term storage. Twenty female C57/BL6 mice and 10 male mice, aged 7–8 weeks, were purchased and raised at the Experimental Animal Center of the Fourth Military Medical University (FMMU, Shaanxi, China) in an SPF environment.

### Neurosphere Culture

The isolation and primary culture of ENCCs originated from fetus mice with a defined gestational age. E11.5 [By E11.5, the ENCCs have colonized through the foregut and midgut and begin to enter the hindgut, where the lesions mainly occurred, thus was chosen for the timing of pathogenesis study (Liu et al., 2015)] mouse guts (from stomach to hindgut) were dissected in L15 medium (Invitrogen, Rockville, MD, United States) and digested with collagenase/dispase (0.2 μg/ml each; 37°C for 15 min) after being washed with Ca^2+^ and Mg^2+^ free PBS. Digested guts were triturated into single cells and filtered through cell strainers (100 μm and then 40 μm, Falcon, BD Biosciences). The cells were resuspended in neural crest medium, DMEM that contained 10% chick embryo extract (SIL, Sussex, United Kingdom), FGF (20 ng/ml, Sigma-Aldrich, St. Louis, MO, United States), EGF (20 ng/ml, Sigma-Aldrich), Retinoic acid (35 ng/ml, Sigma-Aldrich), N2 (1%), B27 (2%, Invitrogen), and β-mercaptoethanol (50 mM, Sigma-Aldrich); the cells were plated into round-bottomed, low-attachment, sterile 6-well plates (Corning, Costar) at a density of 1 × 10^5^ cells/ml. At 3 day intervals, culture medium that contained non-adherent cells was transferred to a new dish and replenished with an equal volume of fresh medium to enable neurosphere formation. The ENCCs at passage two were transferred to culture medium that contained 10% FBS, BMP2 (1 nM, PeproTech), GDNF (50 ng/ml, PeproTech), NRG1 (1 nM, PeproTech) or adenoviruses (1.19 × 10^6^/1 × 10^5^ cells/well) for an additional 48 h for the subsequent functional analyses.

### Human mRNA Microarray Analysis

Three pairs of colon tissues from HSCR ganglionic controls and aganglionic segments were randomly chosen for microarray analysis (this may introduce some bias to the study because HSCR tissue may have differential baseline characteristics such as a different series of transcription factors and growth factors in the micro environment that due to the disease itself). Total RNA in tissue blocks was extracted with Trizol (Invitrogen, United States). Total RNA was further purified by the NucleoSpin^®^ RNA clean-up kit (740.948.250) and then quantified by spectrophotometry; its integrity was measured by agarose gel electrophoresis. Chip hybridization: the cy3-dCTP labeled purified product was mixed with 2X GEx Hyb Buffer and formamide; then, 100 μL of mixed solution was added to the hybridization cassette. It was mounted on the rotor of the hybridization oven (G2545A, Agilent, Santa Clara, CA, United States) and hybridized overnight at 45°C. Chip cleaning and scanning: the chip was removed to Chip Slide Washer8; the cleaned chip was scanned with a chip scanner (G2565CA, Agilent) to obtain a hybrid image. Chip data analysis: hybrid images of extracted data were analyzed using Agilent Feature Extraction software (v10.7). The data were then normalized and analyzed by Agilent GeneSpring v12.0 software (Agilent, Santa Clara, CA, United States). Hierarchical Clustering analysis was applied to present gene expression patterns. The microarray work was performed by KangChen Bio-tech, Shanghai, China.

### Antibodies and Plasmids

The antibodies and dilutions used were as follows: mouse anti-Sox10 antibody (Santa Cruz, immunofluorescence, 1:50); Goat anti-PTPRR antibody (Santa Cruz, immunofluorescence, 1:50); Rabbit anti-GFAP antibody (Abcam Inc., immunofluorescence, 1:500); Rabbit anti-TUJ1 antibody (immunofluorescence, 1:200); Rabbit anti-RET antibody (Promega, immunofluorescence, 1:100); Alexa Fluor^®^ 488 Goat-anti-rabbit IgG (Invitrogen; immunofluorescence, 1:500); Alexa Fluor^®^ 594 Goat-anti-rabbit IgG (Invitrogen, immunofluorescence, 1:100); Alexa Fluor^®^ 488 Goat-anti-mouse IgG (Invitrogen, immunofluorescence, 1:1000); Alexa Fluor^®^ 594 Goat-anti-mouse IgG (Invitrogen, immunofluorescence, 1:100); Alexa Fluor^®^ 488 Donkey-anti-goat IgG (Invitrogen, immunofluorescence, 1:500); Goat-anti-rabbit HRP (Invitrogen, immunoblotting); Rabbit anti-PTPRR antibody (Proteintech Group, immunoblotting, 1:50); and β-actin (immunoblotting, 1:10000).

Adenovirus: PTPRR-shRNA#3/PTPRR-shRNA#5/PTPRR-shRNA#6; PTPRR-ExpRNA; blank-shRNA/blank-ExpRNA were repaired by Cyagen Biosciences, Guangdong, China.

### Western Blot Analysis

The tissues and cells were lysed on ice, according to the instructions. Protease Inhibitor Cocktail (MedChem Express, Monmouth Junction, NJ, United States) was used in the cell lysates to increase the protein stability. After SDS–PAGE, the proteins were transferred to PVDF membranes (0.22 μm, Invitrogen), using a Bio-Rad Semi-Dry Electrophoretic Transfer Cell. Western blot analyses were performed using the corresponding specific antibodies, followed by HRP conjugated IgG antibody. An enhanced chemiluminescence against HRP was used for the visualization of immunoreactive proteins.

### Quantitative Real-Time PCR

Real-time PCRs in a Prism 7500 real-time thermocycler (Applied BioSystems, Foster City, CA, United States) were performed with SYBR Green Ex Taq (Takara), according to the manufacturer’s instructions. The PCR conditions were 95°C for 30 s, followed by 40 cycles of 95°C for 3 s and 60°C for 30 s. All experiments were performed in triplicate. Fold changes in expression were calculated using 2^ΔΔCt^. The primer sequences are provided in Supplementary Table S1.

### Immunofluorescence Analysis

Immunohistochemistry was performed per the instructions, Serial sections (5 μm) of paraffin-embedded samples were deparaffinized and rehydrated with an ethanol gradient. After the inactivation of endogenous peroxidase with 3% H_2_O_2_-methanol at 37°C for 10 min, the sections were washed three times in PBS and blocked with goat serum for 60 min. The sections were subsequently coated with primary antibodies and incubated in a humid box at 4°C overnight. PBS was used instead of antibodies as a negative control. After the addition of fluorescent secondary antibody, the sections were incubated at 37°C for 60 min and then washed three times in PBS.

### EdU Immunochemistry/Cell Proliferation

In this experiment, EdU nuclear staining was used to detect the DNA replication activity of the cells, and the virus-free group was used as a negative control. The blank plasmid group was used as a blank control to detect the proliferation of ENCCs in each group. The specific steps are as follows: Logarithmic growth phase ENCCs were seeded in 6-well plates at 1 × 10^5^ cells per well and incubated for 12 h. The ENCCs were transfected with PBS and downregulated or overexpressed adenovirus according to the group for 48 h. The culture medium that contained the suspension cell pellets was transferred into a 6-well plate preplaced with sterile slides. Then, 200 μl of ENCC complete medium that contained 20 μM EdU was added to each well and incubated for 24 h. One milliliter of methanol was added to each well for 10 min and then washed with PBS for 3 min, 2 times. One milliliter of 0.2% TritonX-100 was added per well to the cell membrane for 10 min and washed with PBS for 3 min, 2 times. One milliliter of Apollo^®^ staining reaction solution was added to each well, after incubating for 30 min at room temperature in the dark; 1 ml of PBS that contained 0.2% TritonX-100 was added to each well and then washed for 10 min, 2 times on a bleaching shaker. One milliliter of Apollo^®^ 567 reaction was added per well, incubated for 30 min at room temperature in the dark, and washed 1∼3 times with 1 ml PBS per well; 1 ml DAPI (1: 100) was added to each well for 5 min and then rinsed twice with PBS.

### Gut Organ Culture

The E11.5 fetal gut was dissected along with its surrounding tissues using an anatomical microscope (Olympus). The gut was attached to a thin strip of filter membrane (Millipore) by pressing a part of the connective tissues to the membrane strip with the tips of forceps. The gut-attached filter paper was suspended with the gut-side down in a 35-mm glass-bottomed dish to avoid direct contact of the gut tissue to the glass bottom. The guts were cultured in DMEM/F12 that contained 10% FBS and penicillin/streptomycin at 37 °C with 5% CO2.

### Whole-Mount IF Staining

Fixed E14.5 gut samples were blocked in PBST (PBS, pH 7.4, with 1% Triton-X 100), 1% cold water fish skin gelatin, and 100 mM glycine with 10% normal serum matching secondary species (Jackson ImmunoResearch). Primary antibodies were incubated overnight in blocking solution at 4°C. Fluorophore-conjugated secondary antibody incubation was performed for 1 h at 25°C. DAPI (100 ng/ml) was subsequently added after the secondary incubation.

### Statistical Analysis

The data were presented as the mean ± standard deviation (SD) from at least three independent experiments. The differences among/between each groups were analyzed with one-way ANOVA or Student’s *t* test. Differences were considered statistically significant when *P* < 0.05(^∗^), *P* < 0.01(^∗∗^). Statistical analyses were performed using SPSS software (SPSS 16.0, Chicago, IL, United States). All statistical tests were two-tails.

## Results

### Decreased PTPRR Expression Is Associated With HSCR

**FIGURE 1:**
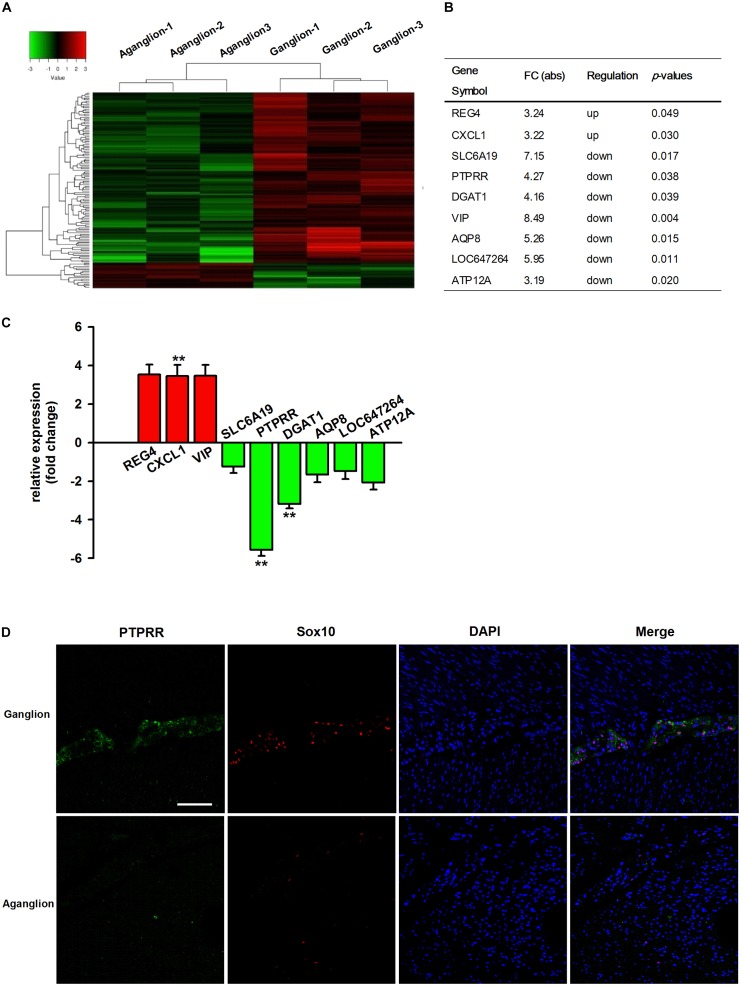
The profile of differentially expressed mRNAs from microarray and qRT-PCR data. **(A)** Hierarchical clustering of the differentially expressed mRNAs in the colon tissues from HSCR aganglionic and ganglionic controls; Red color indicates upregulated mRNAs, and green color indicates downregulated mRNAs (*n* = 3). **(B)** Collection of the top 9 up- or downregulated mRNAs in the colon tissues from HSCR aganglionic compared with ganglionic control tissues; **(C)** The validation of the top 9 differentially expressed mRNAs in the colon tissues from aganglionic compared with ganglionic tissues by qRT-PCR analysis (*n* = 30). **(D)** Confocal assay for PTPRR and Sox10 expression in control or aganglionic colon tissues (*n* = 9). Nuclear staining with 4′,6-diamidino-2-phenylindole (DAPI) is also shown. Aganglion: aganglionic colon tissue. Ganglion: Ganglionic control tissue. Scale bar, 50 μm. ^∗∗^*P* < 0.01, ^∗∗∗^*P* < 0.001.

It has been reported that genetic factors and their regulatory mechanisms may play an important role in the development of HSCR (Bahrami et al., 2018; Sribudiani et al., 2018). To better understand the etiology and pathogenesis of HSCR, we compared the expression differences of mRNA in three pairs of colon tissues from HSCR ganglionic controls and aganglionic segments. The microarray results indicated that 122 mRNAs were differentially expressed (absolute fold change ≥ 2; *P* < 0.05, Figure 1A). Compared with the adjacent ganglionic tissues (data not shown), 16 and 106 mRNAs were up- and downregulated, respectively. The top 9 up- or downregulated mRNAs are listed in Figure 1B. To validate the microarray results, the expressions of the top 9 mRNAs were then validated by qRT-PCR in 30 additional cases. Among them, the change in PTPRR was the most downregulated one with statistical significance (Figure 1C). It suggests that *PTPRR* gene changes are closely related to HSCR. Immunofluorescence staining was subsequently conducted in aganglionic and control colon tissues. There was a deficient positive staining in the lesion tissue, while PTPRR was mainly expressed in the myenteric nerve plexus, which suggests that PTPRR may be involved in the process of intestinal neurodevelopmental disorders that lead to disease.

### PTPRR Is Expressed in Multipotent ENCCs and Downregulated in Neural Progeny

**FIGURE 2:**
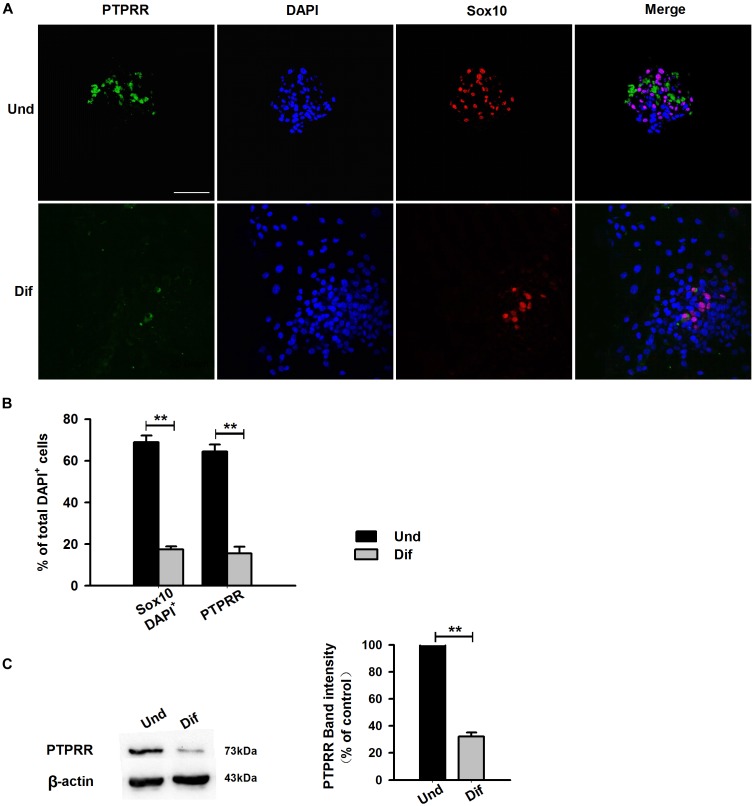
Expression of PTPRR in ENCCs compared with neural progeny in maturation medium. Confocal images of immunofluorescence expression of PTPRR in neural precursor ENCCs (**A**, the upper panel) and their neural progeny induced by maturation medium (**A**, the bottom panel). Cell nuclei were counterstained with 4′,6-diamidino-2-phenylindole (blue). **(B)** Quantification of PTPRR immunofluorescence from undifferentiated ENCCs (abb: Und) compared with their differentiated neural progeny (abb: Dif). **(C)** Western blotting was also conducted to detect the protein levels of PTPRR in different groups. *N* = 8. ^∗∗^*P* < 0.01, ^∗∗∗^*P* < 0.001. Scale bars, 25 μm.

**FIGURE 3:**
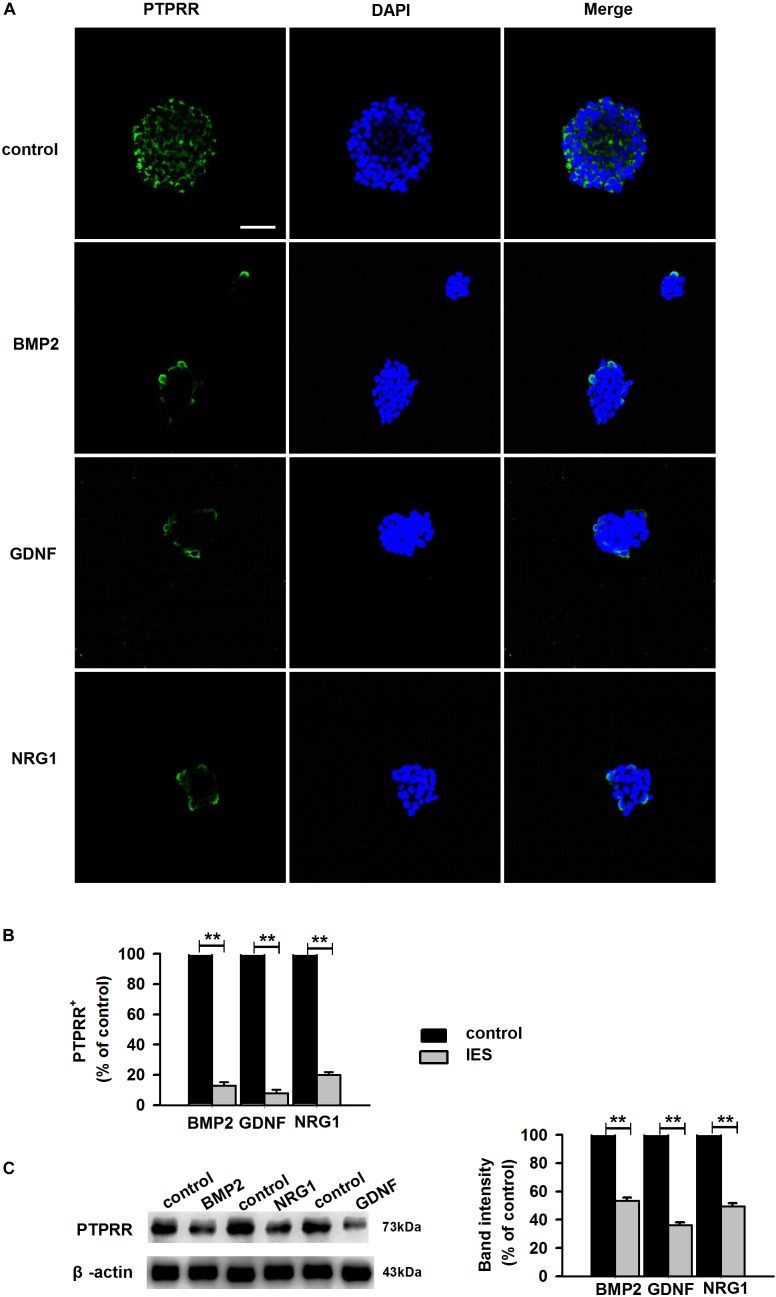
Expression of PTPRR in ENCCs exposed to GDNF/BMP2/NRG1. Confocal images of immunofluorescence expression of PTPRR in neural precursor ENCCs (**A**, the upper panel) and their neural progeny induced by GDNF, BMP2 or NRG1 (**A**, the other panel). Cell nuclei were counterstained with 4′,6-diamidino-2-phenylindole (blue). **(B)** Quantification of PTPRR immunofluorescence from undifferentiated ENCCs (abb: Und) compared with their differentiated neural progeny (abb: Dif) (*n* = 10). **(C)** Western blotting was also conducted to detect the protein levels of PTPRR in each group. *N* = 10. ^∗∗^*P* < 0.01, ^∗∗∗^*P* < 0.001. Scale bars, 25 μm.

Protein tyrosine phosphatase receptor-type R was mainly expressed in the enteric plexus and with a consistent distribution pattern with Sox10 from control colon tissues (Figure 1D upper panel), which prompted us to further investigate the cellular localization and function of this protein. Immunocytochemistry results of the PTPRR expression in primary cultured mouse ENCCs demonstrated that most of these cells were positive for the neural progenitor markers Sox10 and RET (Supplementary Figure S1A) and can also generate neurons and glia (Supplementary Figure S1B). It has been reported that Sox10 is a specific transcription factor marker for neural precursor cell ENCCs (Bondurand et al., 2006), and RET is a proto-oncogene encoded by the RET gene, which is mainly expressed in glial and neuronal precursor cells (Kjaer et al., 2010) These results support the idea that PTPRR is expressed by multipotent ENCCs. According to the previous findings (Kelsh, 2006), there was a significant reduction of ENCCs (Sox10+-DAPI+) in the maturation medium that contained 10% fetal bovine serum (FBS) due to differentiation (Figure 2A, bottom panel) into various neural progenies. The reduced expression levels of PTPRR in the neural progeny derived from the ENCCs compared to the multipotent ENCCs were verified by immunocytochemistry and Western Blotting. The percentage of PTPRR positive cells was 15.6 ± 3.2% (*P* < 0.01) in the neural progeny and 64.5 ± 3.3% (*P* < 0.01) in the multipotent ENCCs (Figure 2A,B), whereas there was a 72.1 ± 2.6% (*P* < 0.01) reduction in the basal values of the cell protein expression (Figure 2C). Moreover, the differentiation of ENCCs can be promoted by specific instructive extracellular signals (IES), such as GDNF (Uesaka et al., 2013), BMP2 (Fu et al., 2006), and NRG1 (Paratore et al., 2001; Supplementary Figure S2). We assumed that these factors induced the downregulation of PTPRR expression in advance of this overt differentiation. The application of BMP2 (1 nM), GDNF (50 ng/ml) or NRG1 (1 nM) caused an extinction of PTPRR expression in 92.9% ± 2.4% (*P* < 0.01), 95.6% ± 2.5% (*P* < 0.01) and 88.6% ± 1.9% (*P* < 0.01) of ENCCs, respectively (Figure 3A,B), in accordance with the results in western-blot analysis (Figure 3C).

### PTPRR Maintains Neurogenic and Proliferative Potential in ENCCs

**FIGURE 4:**
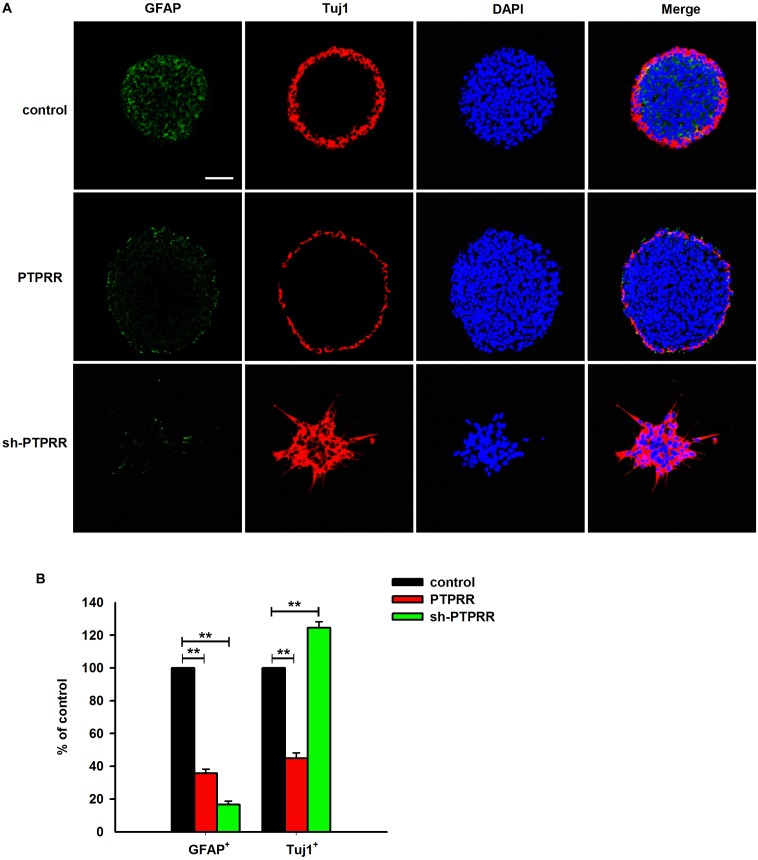
PTPRR preserves neurogenic potential of ENCCs. PTPRR preserves glial (marked as GFAP) and neuronal (marked as Tuj1) differentiation potentials in individual fluorescence channels from the triple-labeled fields shown in **(A)**. **(B)** Quantification of neuronal and glial immunofluorescence from PTPRR up- (PTPRR) and downregulation (sh-PTPRR) ENCCs (*n* = 10). Values are normalized to control. ^∗∗^*P* < 0.01. Scale bars, 25 μm.

**FIGURE 5:**
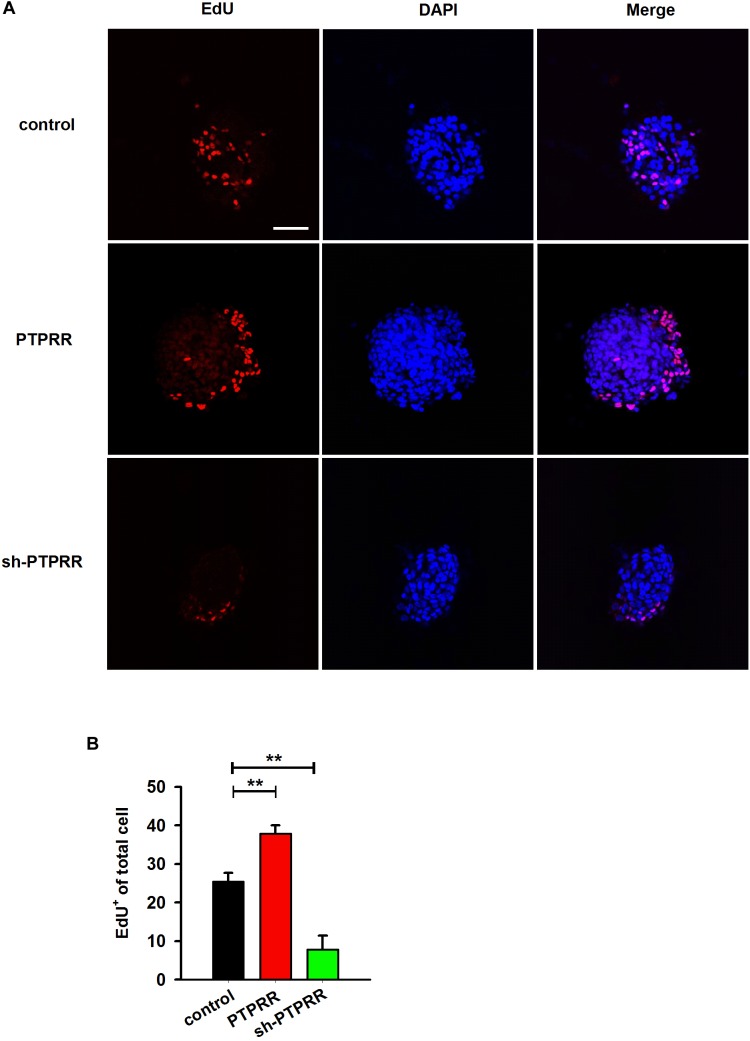
PTPRR preserves proliferative potential of ENCCs. **(A)** EdU immunoreactivity after 24 h incubation with 20 μM EdU in PTPRR up- (PTPRR) and downregulation (sh-PTPRR) ENCCs (*n* = 10) immediately prior to collection. Single optical sections show adenoviruses infected ENCCs that had incorporated EdU. **(B)** Quantification of DAPI^+^- EdU^+^ double-positive cells within DAPI^+^ cells in each group (*n* = 10). ^∗∗^*P* < 0.01. Scale bars, 25 μm.

The stable expression of PTPRR in neural precursors and rapidly downregulated by factors that promote differentiation raised a question of whether it is required for the maintenance of the multipotent potential. Thus, we supposed that the regulation of the PTPRR expression would influence the differentiation potential in ENCCs. To verify this hypothesis, we infected ENCCs with adenoviruses to up- or downregulate PTPRR (Supplementary Figure S3). After 24 h to permit the expression of the virally encoded transgene, the upregulation of PTPRR caused a significant loss of gliogenic and neurogenic differentiation, as reflected in a lack of both GFAP and Tuj1 expression. Moreover, most ENCCs differentiated into neurons after PTPRR downregulation (Figure 4A,B). These results confirm our hypothesis that PTPRR maintains the gliogenic and neurogenic potential of ENCCs. Furthermore, according to the EdU fluorescent chemical staining (Figure 5A), the EdU^+^ cells in the PTPRR knockdown group was significantly reduced by approximately 20% ± 4.1% (*P* < 0.01) (Figure 5B). Thus, the expression level of the PTPRR gene is closely related to the proliferation ability of ENCCs.

### PTPRR Inhibits Overt Neuronal Differentiation in ENCCs

**FIGURE 6:**
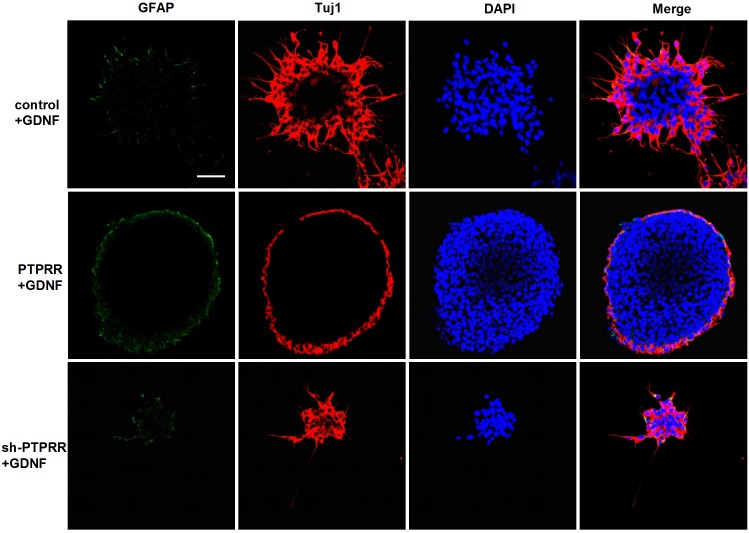
PTPRR inhibits GDNF-induced neuronal differentiation in ENCCs. Immunofluorescence for glial marker GFAP and neuronal marker Tuj1 were used to assess the differentiation capacity of ENCCs after adenovirus transduction, assessed via exposure to 50 ng/ml GDNF. The neurospheres maintained undifferentiated patterns with reduced Tuj1 expression in PTPRR overexpression group. There were apparent differentiated patterns and mainly Tuj1 positive cells in down regulation shRNA group. *N* = 8. Scale bars, 25 μm.

The previous study showed a significant effect of GDNF-induced differentiation of ENCCs on the PTPRR protein expression (Figure 3A–C), which suggests that PTPRR protein may be involved in the GDNF-induced differentiation of ENCCs. In this part of the study, GDNF-induced differentiation of ENCCs was observed when PTPRR was up- or downregulated. The staining results showed that compared with the negative control and the blank control, the GDNF-treated ENCCs in the PTPRR overexpressing group remained undifferentiated, since the positive staining of Tuj1 was significantly reduced. In the PTPRR knockdown group, the spheres showed a clear differentiation status after GDNF treatment (Figure 6). Moreover, these differentiated cells were mostly positive for Tuj1 staining. In addition, there was no significant staining difference of GFAP in each group, which accorded with the neuronal directional differentiation capacity of GDNF. This result indicates that PTPRR gene expression affects the effect of GDNF-induced differentiation of ENCCs, thereby maintaining the undifferentiated status and differentiation potential as neural precursor cells.

### Expression of PTPRR Maintains Migration of ENS

**FIGURE 7:**
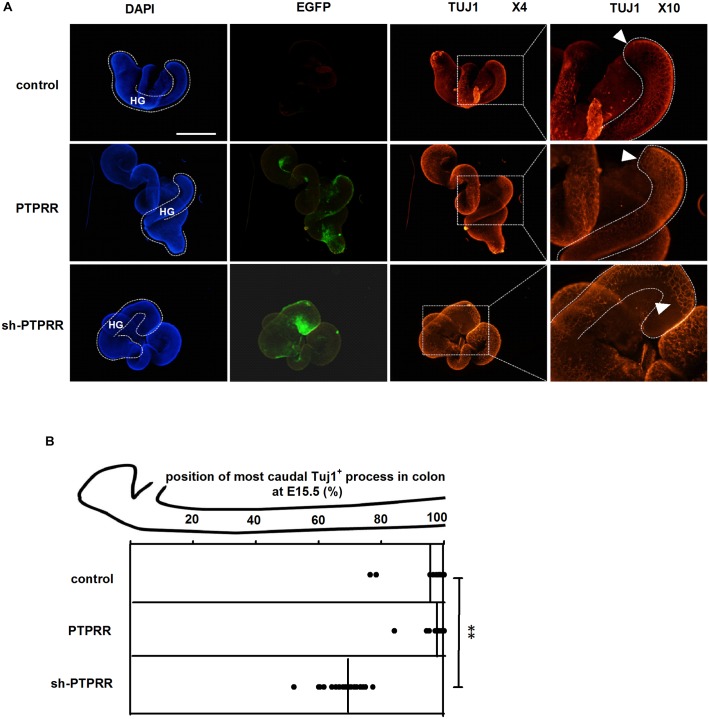
PTPRR downregulation inhibited ENS development in whole-mount gut culture. **(A)** Dotted lines denote outline of hindgut. The ENS colonization within each E15.5 colon of the most caudal region was visualized by the neuronal marker Tuj1. The Tuj1 positive immunofluorescence of hindgut (HG: shown as white curve in DAPI column) migrated to the most caudal region both in control and PTPRR groups, whereas there was an arrested migration (the last Tuj1 column: display by white arrowheads) in PTPRR downregulation gut. **(B)** The position within each E15.5 colon of the most caudal neuron (ascertained by Tuj1) is plotted for each treatment (thick lines denote mean).^∗∗^*P* < 0.01, *n* = 10. Scale bars, 1 mm.

Enteric nervous system colonization of the gut depends on the proper neural crest proliferation and differentiation. To determine whether PTPRR is required for efficient gut colonization, we infected fetal gut with adenoviruses in a 72 h whole gut culture from E11.5 to E14.5, the period in which enteric neurons migrate from the midgut to the most caudal region. There was a distinct GFP positive immunofluorescence in the adenovirus transduction gut (Figure 7A). The ENS marked by Tuj1 red immunofluorescence of the hindgut migrated to the whole gut in both the normal and blank control groups, whereas there was an arrested development (Tuj1 deficit expression toward the end of hindgut) in the PTPRR knockdown gut (Figure 7A,B). This observation indicates that PTPRR is required for ENS migration and enable complete innervation of the gut.

### Inhibition of Neurogenesis of GNDF by PTPRR Is Correlated With Its Repression of pERK

**FIGURE 8:**
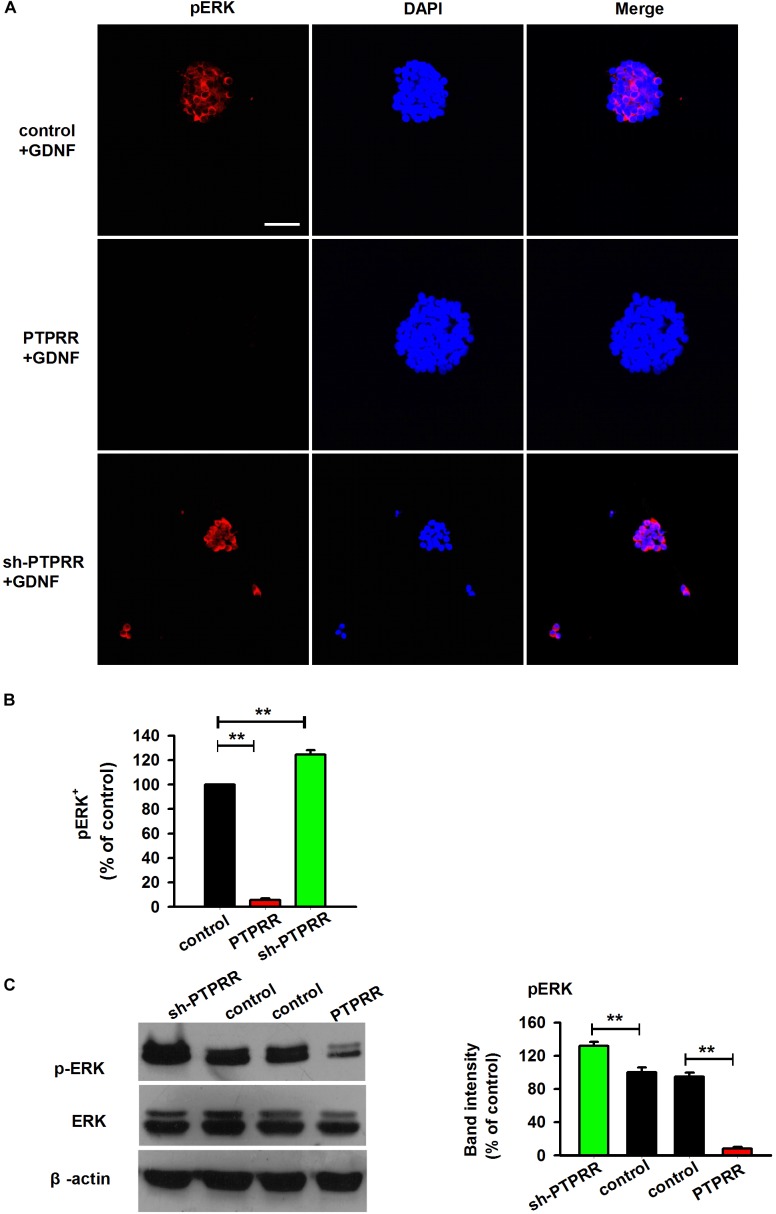
Inhibition of neurogenesis of GNDF by PTPRR is correlated with the repression of pERK. The expression of pERK activated by GDNF significantly decreased as reflected by immunofluorescence **(A,B)** or western-blot **(C)** analyses after genetic modulation in PTPRR Exp-RNA group. ^∗∗^*P* < 0.01, *n* = 8. Scale bars, 25 μm.

In the peripheral enteric nervous system, the MAPK pathway is a key signaling pathway during the GDNF-mediated directional differentiation of ENCCs. To further clarify the potential mechanism by which PTPRR maintains the multipotency of neural progenitor cells, we examined the phosphorylation level of ERK1/2 (Milosevic et al., 2006; Chen et al., 2007) a key signaling molecule in the activation of the GDNF-mediated MAPK pathway (Schaeffer and Weber, 1999; Miloso et al., 2008), under the pretreatment of the PTPRR gene. The results showed that the GDNF-activated ERK1/2 expression was significantly decreased as reflected by western-blot (Figure 8C) or immunofluorescence analyses (Figure 8A,B) in PTPRR overexpressed cells. These findings suggest that PTPRR may play a role in the maintenance of the multipotency of neural precursor cells by negatively regulating the activation of the MAPK pathway.

## Discussion

Hirschsprung disease is a multifactorial disease, and variations in its phenotype and severity are caused by a combination of genetic and environmental factors. To better elucidate the molecular mechanisms underlying this disease, a large number of studies are devoted to the exploration of susceptibility genes and related cellular and molecular mechanisms. To date, mutations in approximately 20 genes have been identified in HSCR patients (Sribudiani et al., 2018). Among these, *RET* is considered the major susceptibility gene for HSCR disease. In human, mutations in *RET* account for 50% of familial cases of HSCR and up to 35% of sporadic cases (Sribudiani et al., 2018). Moreover, approximately 5% of HSCR cases carry heterozygous mutations in *EDN3, EDNRB* and the endothelin converting enzyme *ECE1* (Heanue and Pachnis, 2007; Bahrami et al., 2018). In recent years, new susceptibility genes and modified genes such as *RELN*, *GAL* and *GAP43* have been identified. However, due to the complexity of ENS development, the study of the etiology of HSCR needs to be comprehensively analyzed from multiple perspectives.

In the present study, a microarray screening was performed using three pairs of HSCR ganglionic and aganglionic tissues. Among the top 9 dysregulated mRNAs, PTPRR was the most downregulated, which was confirmed by qRT-PCR in 30 other cases, revealing an association between the downregulated PTPRR expression and HSCR. Our subsequent histological results further verified the downregulation of PTPRR in HSCR aganglionic segments. The main distribution in the ganglion plexus of ganglionic tissue suggests that PTPRR may be involved in the normal development of ENS. These findings indicate the potential clinical application of PTPRR as a candidate biomarker for the detection of HSCR. According to the current knowledge, the *PTPRR* gene encodes a transmembrane protein known as protein tyrosine phosphatase receptor type R. It may be of particular relevance to the motor coordination function of the central nervous system and may be involved in oncogenesis in the digestive system, as gene knockout mice display an ataxia phenotype while PTPRR are epigenetically silenced in colorectal carcinoma (Menigatti et al., 2009). Further studies have shown that PTPRR is predominately expressed in adult mice cerebellum Purkinje cells, and involved in the function of the central nervous system by regulating ERK1/2 phosphorylation (Schmitt et al., 2009).

The results of our study reveal that PTPRR is expressed in ENCCs and functions as a progenitor maintenance factor that is involved in normal ENS development. A range of findings imply that a certain number of neural precursor cells is needed for the normal development of ENS. As Sox10 is the most well studied factor with a key role in maintaining ENCCs in a proliferative and undifferentiated status (Miyahara et al., 2011). In *Sox10*-null mice, ENS progenitors exhibit a smaller pool size (Britsch et al., 2001), and the number of neurons in the distal intestine of *Sox10* heterozygous mice is reduced (Paratore et al., 2002). Similarly, the RET pathway has been implicated in normal ENCC proliferation and survival. Complete knockout of the *RET* gene results in substantial apoptosis of intestinal precursor cells, resulting in incomplete colonization of ENCCs in the intestine (Lai et al., 2017). In accordance with the previous studies, a significant reduction of ENCCs was observed in condition due to differentiate into various neural progeny. Furthermore, in our genetic modulation study, an apparent differentiated pattern and significantly decreased EdU-positive immunofluorescence were observed in PTPRR down-regulated ENCCs. These data support the notion that PTPRR may ensure a certain number of neural precursor cells by inhibiting ENCC differentiation and maintaining ENCC proliferation. Furthermore, to assess the purported impact of PTPRR on the development of ENS, we evaluated the migration of ENS during embryonic development. Consistent with our assumption, the data from organ culture studies showed that PTPRR downregulation inhibited ENS development *in vivo*.

Furthermore, this gene was demonstrated to be involved in the process by which GDNF induces the differentiation of precursor cells. Studies have shown that the ERK1/2 pathway is an important signaling pathway for specific instructive extracellular signal factors to mediate nervous system development (Milosevic et al., 2006; Miloso et al., 2008). The activation and maintenance of MAPK/ERK1/2 promote the proliferation and migration of neural precursor cells, and a low level of activation or inactivation of this signaling pathway is essential for ENS precursor cells to remain undifferentiated. Uesaka et al. (2013) have suggested that the possibility that ERK is at a low level of activation of GDNF signaling pathway, by which precursors maintaining their progenitor state, is inhibited by negative regulators. Our study explored the mechanism by which PTPRR maintained the multipotency of ENS precursor cells and detected the MAPK pathway activation by GDNF under gene-regulated precursors. It was found that pERK1/2 expression level (the key signaling molecule of the MAPK pathway) in ENS precursor cells was significantly decreased after the upregulation of PPTRR expression, while it was significantly increased after the downregulation of PPTRR expression. However, no significant change was observed in expression of p-JNK and p-p38 (results not shown). These findings indicate that PTPRR may play a role in maintaining the multipotency of ENS precursor cells by negatively regulating the MAPK/ERK1/2 pathway.

In summary, the *PTPRR* gene and its encoded protein have the ability to maintain the proliferation and multi-differentiation of gut neural progenitor cells and ultimately ensure the normal development of ENS. Moreover, it may play a role in inhibiting the overt differentiation of intestinal neural precursor cells by negatively regulating the MAPK/ERK1/2 pathway. These functions are closely associated with the reduction or absence of HSCR enteric neural progenitor cells and subsequently differentiated ganglion cells.

## Conclusion

To conclude, the differential expression of PTPRR was related to the proliferation and differentiation of ENCCs, which confirmed the role of PTPRR in maintaining the multipotency of ENS precursor cells, established PTPRR proteins as negative regulators of MAPK/ERK1/2 signaling cascades. Such direct evidence of ENCC multipotency maintaining may further elucidate understanding of ENS development, thus providing insight into the pathophysiology of HSCR.

## Ethics Statement

This study was approved by the Ethics Committee of The Affiliated Children Hospital of Xi’an Jiaotong University (Shaanxi, China) and the guardians of all subjects involved in the study signed written informed consent. All animal protocols were approved by the Committee for Ethical Use of Experiments Animals of the Fourth Military Medical University in Xi’an, China.

## Author Contributions

JT, CZ, and ZT performed the research. BW, ZS, and XJ designed the research study. YL analyzed the data. YP collected clinical specimens. JT and CZ wrote the manuscript. All authors approved the final manuscript.

## Conflict of Interest Statement

The authors declare that the research was conducted in the absence of any commercial or financial relationships that could be construed as a potential conflict of interest.
