# Supplementary material for: Downregulation of Protein Tyrosine Phosphatase Receptor Type R Accounts for the Progression of Hirschsprung Disease
Source: Front Mol Neurosci. 2019 Apr 10;12:92. doi: 10.3389/fnmol.2019.00092 (PMC6468927; doi:10.3389/fnmol.2019.00092)
Supplement: Supplementary file 1 [file Data_Sheet_1.ZIP › supplimentary/Supplementary material.docx]

**Supplementary material**

**Figure S1 The expression of PTPRR in multipotent ENCCs.** Confocal images of immunofluorescence expression of PTPRR in neural precursor ENCCs (**A**, marked by RET and Sox10) and the progeny of multidirectional differentiation ENCCs induced by standard culture conditions (**B**, the bottom panel, GFAP: glial marker, Tuj1: neuronal marker). Cell nuclei were counterstained with 4´,6-diamidino-2-phenylindole (blue). N=6. Scale bars, 25 µm.

**Figure S2 Differentiation of neural progenitor promoted by instructive extracellular signals (IES).** Immunofluorescence of glial marker GFAP and neuronal marker Tuj1 was used to assess the differentiation status of ENCCs after exposure to 50 ng/ml GDNF, 1 nM BMP2 or 1 nM NRG1. N=8. Scale bars, 25 µm.

**Figure S3 Transfection efficiency of adenoviruses in ENCCs. (A)** Fluorescence image for ENCC neurospheres after adenovirus transfection (n=6). **(B,C)**. Western blotting was conducted to detect the protein levels of PTPRR in each group. The data are shown as the mean ± SD. ***P*＜0.01. N=8. Scale bars, 100 µm.

**Table S1 Primer sequences used for qRT-PCR.**
