# Supplementary figures and images for: Downregulation of Protein Tyrosine Phosphatase Receptor Type R Accounts for the Progression of Hirschsprung Disease
Source: Front Mol Neurosci. 2019 Apr 10;12:92. doi: 10.3389/fnmol.2019.00092 (PMC6468927; doi:10.3389/fnmol.2019.00092)

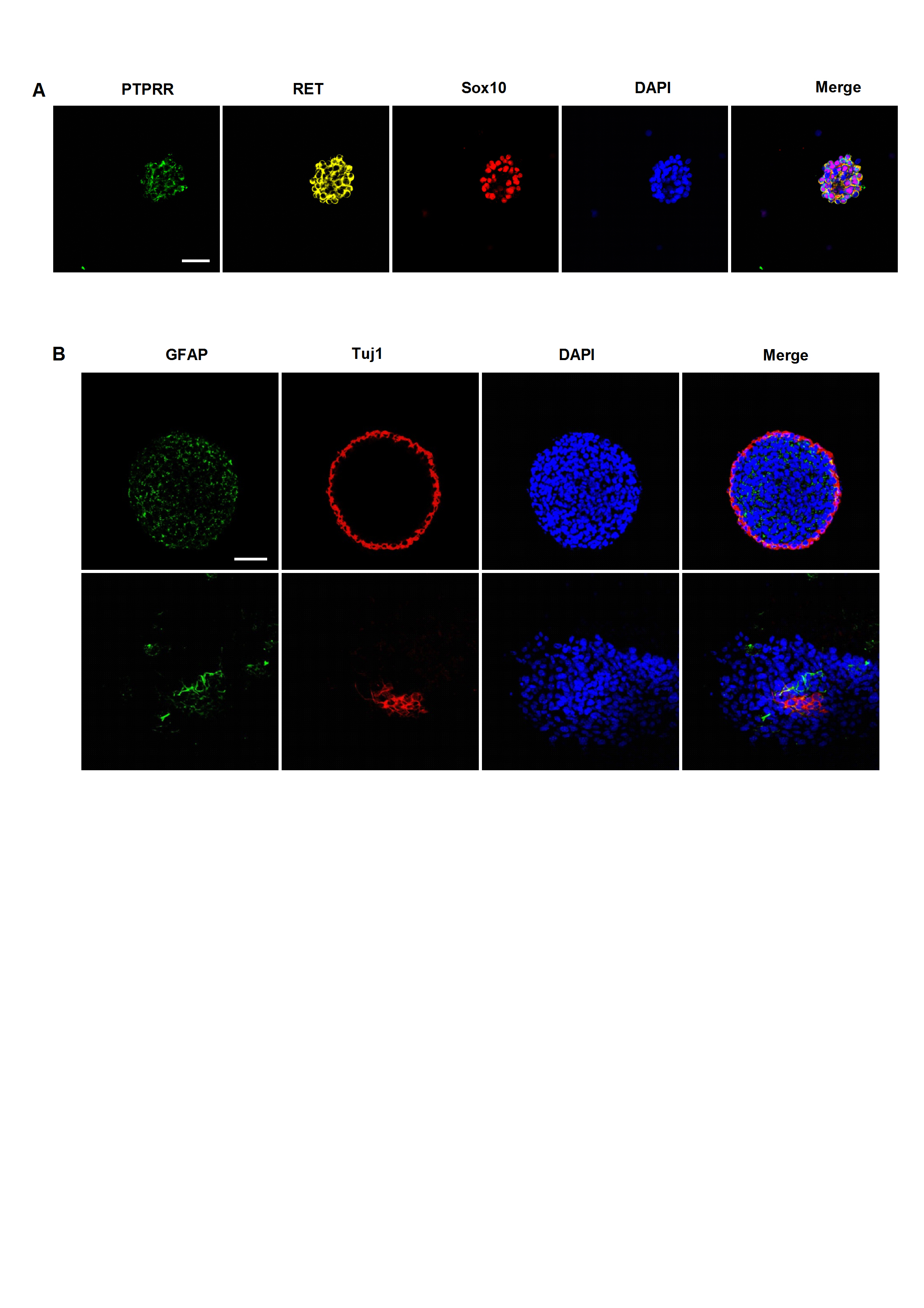

Supplement: Supplementary file 1 [file Data_Sheet_1.ZIP › supplimentary/fig S1.JPG]

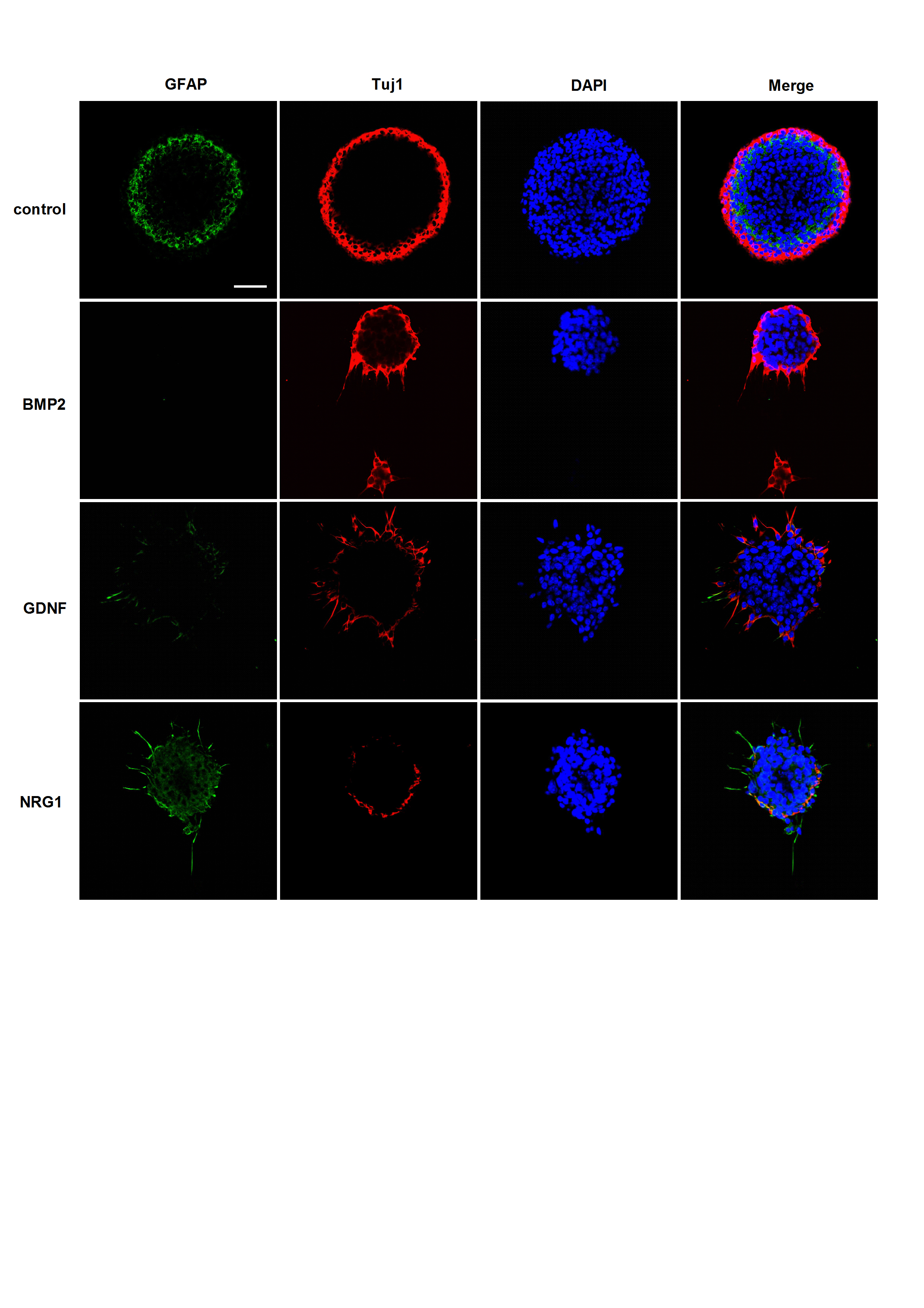

Supplement: Supplementary file 1 [file Data_Sheet_1.ZIP › supplimentary/fig S2.JPG]

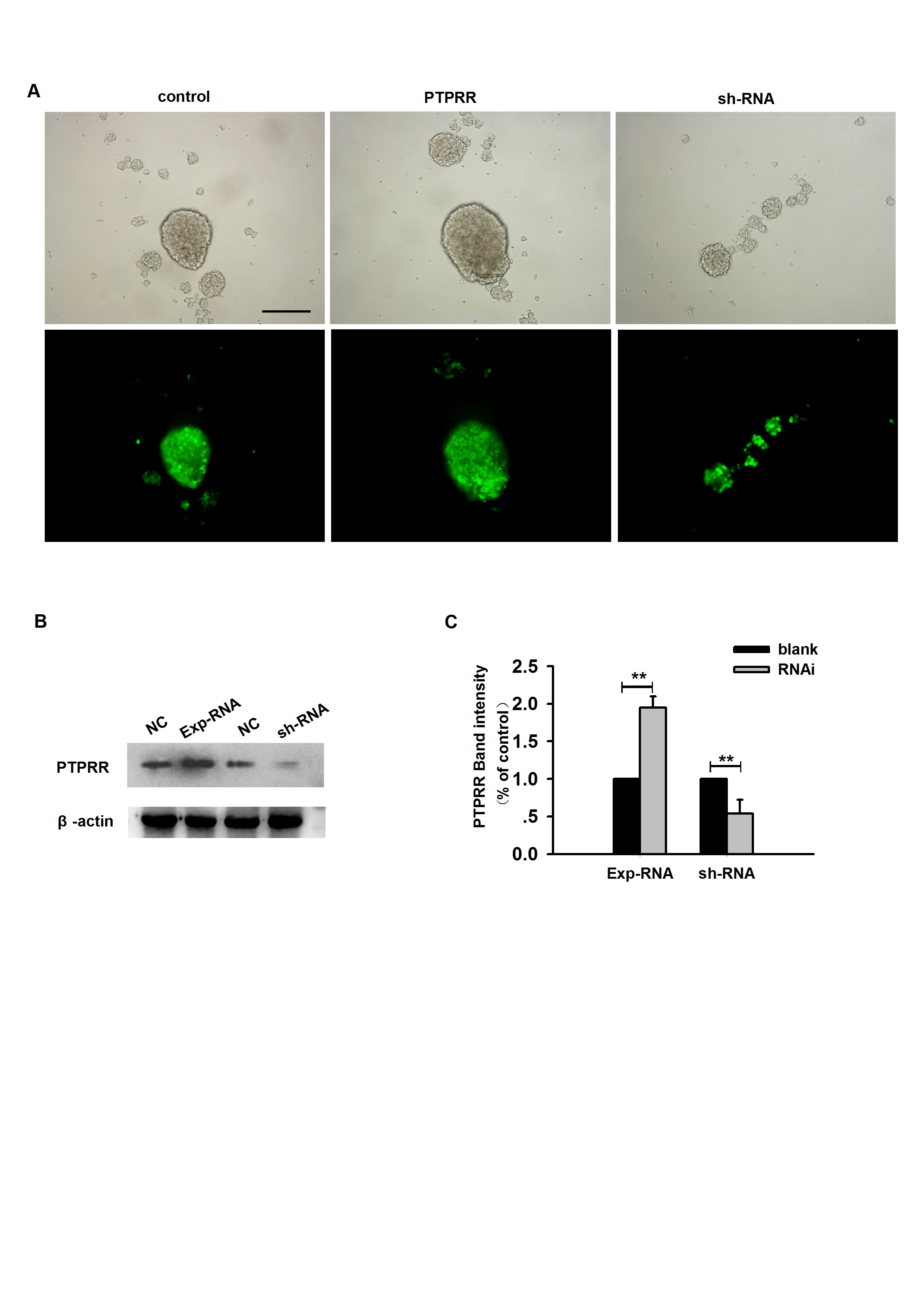

Supplement: Supplementary file 1 [file Data_Sheet_1.ZIP › supplimentary/fig S3.JPG]

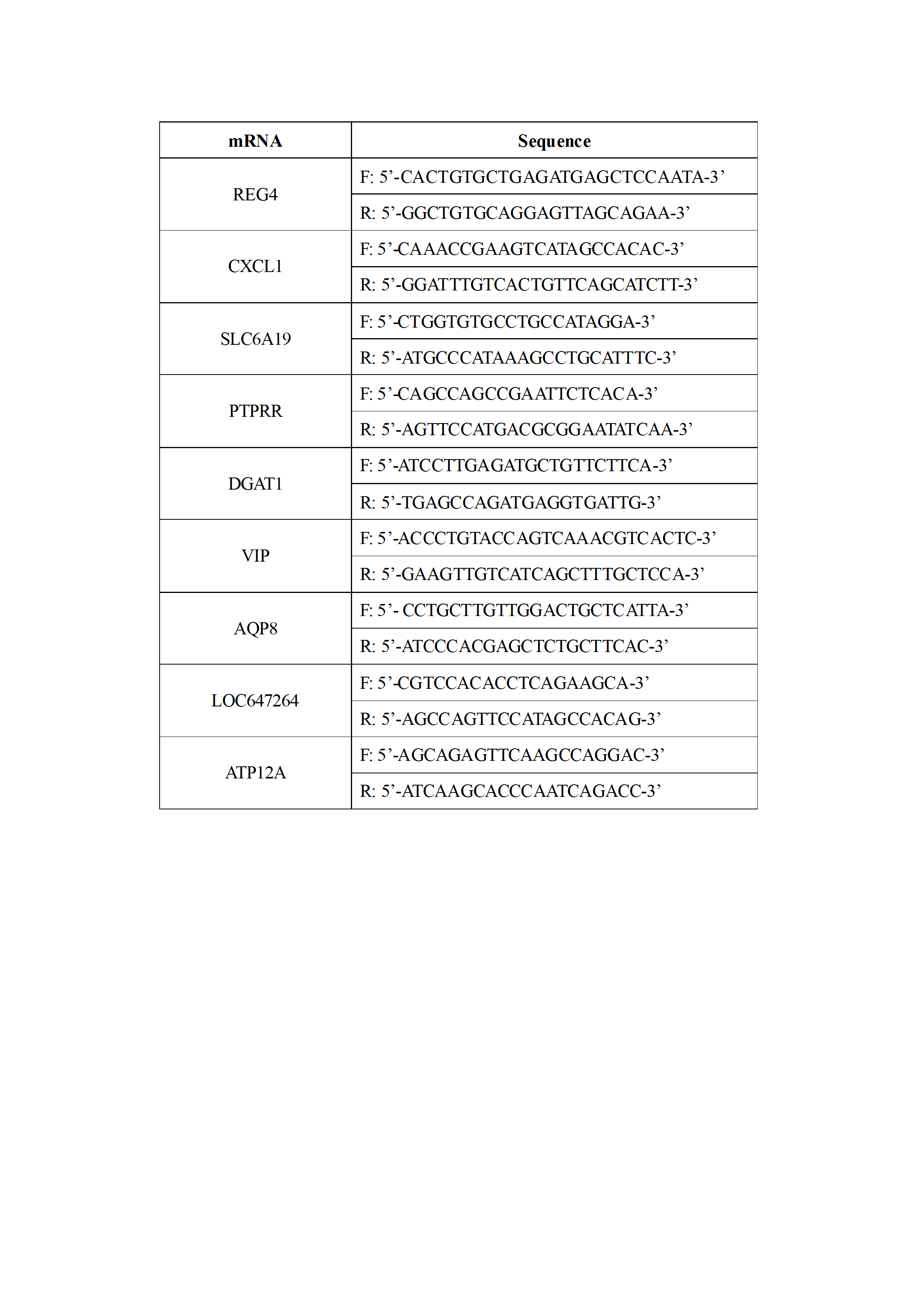

Supplement: Supplementary file 1 [file Data_Sheet_1.ZIP › supplimentary/table S1.JPG]
